# Reengineering Indoor Air Quality Monitoring Systems to Improve End-User Experience

**DOI:** 10.3390/s24082659

**Published:** 2024-04-22

**Authors:** Radu Nicolae Pietraru, Adriana Olteanu, Ioana-Raluca Adochiei, Felix-Constantin Adochiei

**Affiliations:** 1 Faculty of Automatic Control and Computers, National University of Science and Technology Politehnica Bucharest, 313 Splaiul Independentei, 060042 Bucharest, Romania; adriana.olteanu@upb.ro; 2 Emil Palade Center of Excellence for Young Researchers, Academy of Romanian Scientists, Ilfov 3, 050044 Bucharest, Romania; ioana.adochiei@mta.ro (I.-R.A.); felix.adochiei@upb.ro (F.-C.A.); 3 Faculty of Electrical Engineering, National University of Science and Technology Politehnica Bucharest, 313 Splaiul Independentei, 060042 Bucharest, Romania

**Keywords:** indoor air quality, manual ventilation, air quality monitoring systems, IoT monitoring, advanced air quality analysis, end-user experience

## Abstract

This paper presents an indoor air quality (IAQ) monitoring system designed for a better end-user experience. The monitoring system consists of elements, from the monitoring sensor to the monitoring interface, designed and implemented by the research team, especially for the proposed monitoring system. The monitoring solution is intended for users who live in houses without automatic ventilation systems. The air quality sensor is designed at a minimum cost and complexity to allow multi-zone implementation without significant effort. The user interface uses a spatial graphic representation that facilitates understanding areas with different air quality levels. Presentation of the outdoor air quality level supports the user’s decision to ventilate a space. An innovative element of the proposed monitoring interface is the real-time forecast of air quality evolution in each monitored space. The paper describes the implementation of an original monitoring solution (monitoring device, Edge/Cloud management system, innovative user monitoring interface) and presents the results of testing this system in a relevant environment. The research conclusions show the proposed solution’s benefits in improving the end-user experience, justified both by the technical results obtained and by the opinion of the users who tested the monitoring system.

## 1. Introduction

In the urban environment, people spend up to 90% of their time inside buildings, thus exposing themselves to a health threat identified as sick building syndrome (SBS) [1]. Indoor air pollution has been cataloged as one of the top five risks to public health. The long-term monitoring of polluting compounds inside buildings benefits the health and well-being of people who live and work indoors [2]. There is a strong association between poor air quality index (AQI) conditions and respiratory, cardiovascular, and dermatological diseases [3]. The study of the effects of indoor air quality on human health can be traced in the specialized literature for over 40 years and covers effects on cognitive power, psychological effects, effects on the urological system, effects on the ENT system, and the human nervous system [4].

Indoor air quality (IAQ) is affected by pollutants such as dust (particulate matter—PM), volatile organic compounds (VOC), carbon monoxide (CO), nitrogen oxides (NO_x_), carbon dioxide (CO_2_), ozone (O_3_), radon, biological allergen compounds, and other microorganisms [1,2,5]. PM and VOC are two pollutants with a significant effect on AQI [3,5], and they are also the parameters monitored and forecasted as evolutions by our proposed solution. The primary sources of indoor air pollution are current human activities (cooking, cleaning, smoking, repair activities), emissions from materials and objects inside the building (furniture, floor material, paints, perfumes, and indoor air fresheners), and the heating of the interior space if it is based on combustion (gas, coal, oil) and if the combustion source is in the inhabited perimeter [6].

The limit concentrations of polluting agents for air quality assessment are presented and regulated in multiple international and governmental standards and regulations [7]. Still, there are several challenges related to the area of applicability, the polluting agents considered, whether it refers to outside air, indoor air, or both, the pollutant measurement methodology, the obligation to apply, and the purpose of the application (certification of construction or ventilation installations versus the evaluation of human well-being and health). In [8], an analysis of the priority of application of the standards is made, identifying the most priority level as the national legislation in the field and going from mandatory to recommended, good practice regulations, administrative regulations, technical guides, and specific standards. From this perspective, the 2021 WHO Guide for Air Quality [9] is an important document that will probably guide the evolution of the rest of the standards and legislation in the field around the globe this decade.

Due to the complexity of the air quality evaluation problem, the user may have difficulty evaluating the air quality in his living space. Several simplifying approaches hide the complexity of evaluating several concentrations of polluting agents in a single indoor air quality index represented according to severity with suggestive colors, both concepts being commonly used to evaluate outdoor air quality. In [10], an indoor air quality evaluation index with four colored levels is proposed using measured concentrations for SO_2_, NO_2_, CO, O_3_, PM2.5, and PM10.

One of the solutions to reduce indoor air pollution is the implementation of efficient ventilation (starting from the standards and best practice guidelines). However, in many developing or underdeveloped regions, the implementation regulations are still in the development phase or do not exist [5]. Studies show adequate ventilation can combat pollution with various agents (CO_2_, VOC). However, there are still situations in which existing studies reach their limits of validity for reasons related to the climate and the specifics of buildings or pollutants [6]. For example, ensuring IAQ in heritage buildings cannot be achieved by ordinary technical methods and requires a multidisciplinary approach [11]. The work of [12] demonstrates that the usual ventilation policies can fail to ensure a healthy environment in crowded spaces, such as classrooms and teaching laboratories. The differences in the IAQ assessment also affect how the various ventilation standards are effectively implemented [13,14].

This paper proposes an IoT architecture (and presents a prototype implementation) for indoor air quality monitoring for buildings without an automatic ventilation system (and where one cannot be installed). In these situations, ventilation and IAQ insurance are the responsibility of the people who live in that building, and the proposed solution meets their needs. The monitoring system uses a state-of-the-art integrated sensor capable of measuring the following environmental and air quality parameters: ambient temperature and humidity, the concentration index of volatile organic compounds (VOC), the concentration index for nitrogen oxides NO_x_, and the concentration in the air of particulate matter (PM) with diameters below 1.0, 2.5, 4.0, and 10 μm. Organic compounds from n-hexane up to and including n-hexadecane are considered volatile organic compounds. NO_x_ represents the sum of NO and NO_2_ and contributes to the oxidation of VOCs, generating OVOCs (oxygenated volatile organic compounds) and other oxidizing compounds such as O_3_.

The proposed monitoring system (presented in Section 3 of the paper) aims to add the following significant improvements to user support:

- The data reported to the user are processed and visually represented so that they are easy to understand.
- The proposed monitoring architecture correlates the data measured from inside and data from the municipal air quality monitoring networks and offers the user an efficient ventilation perspective.
- The architecture proposed in this paper introduces an indoor air quality forecasting component that can help the user to mitigate the decrease in air quality.

## 2. Related Works

The research areas related to our work and the contributions outlined above are indoor air quality (VOC, NO_x_, PM) parameters and their implications for human health. This comprehensive review thoroughly examines the factors impacting air pollution in residential and commercial buildings [5].

The proliferation of research on IAQ monitoring systems has been remarkable in recent years. Despite the surge in research, a common thread emerges across most projects: the widespread adoption of well-established air-quality monitoring parameters and communication technologies.

Numerous reviews delve into the cutting-edge advancements in Internet of Things (IoT)-based IAQ monitoring systems, encompassing sensor technologies, microcontroller platforms, system architectures, and connectivity solutions. They also examine the significant impact of communication technology and the engineering design aspects of indoor air quality control systems [15,16,17,18,19,20].

A plethora of research papers have explored the complexities of IAQ monitoring systems. These studies have addressed various aspects of IAQ monitoring, including sensor technology, data collection methodologies, data analysis techniques, and system implementation approaches. Table 1 presents the state of the art of existing IAQ monitoring systems and gives an overview of a selection of qualification studies conducted from 2018 to 2023 in the field. Notice that studies relate only to ventilation systems, multi-zone technology, edge-computing capabilities, monitored parameters, index reports, exterior data, real-time forecasting, user interface, and type and cost monitoring of monitoring devices.

From the prediction of the evolution of air quality indicators point of view, we highlight two significant studies. The authors of [31] comprehensively analyzed papers on air quality prediction using machine learning. They evaluated these papers based on 14 critical criteria, encompassing the study’s motivation, the type of modeling approach employed (estimation or forecasting), the machine-learning algorithms utilized, the methodology used by the authors, the nature of the predicted parameter (specific pollutants or overall air quality), the geographic location of the study, dataset characteristics (period, number of monitoring stations, and instance count), dataset specificity in terms of predictive attributes, evaluation methods and performance metrics (accuracy, mean absolute error, root mean square error, and coefficient of determination), and the computational cost of the proposed methods. Next, Xu and Ren [32] analyzed a diverse range of predictive models that have emerged for air pollution concentration prediction, including autoregressive integrated moving average models, support vector machines, multiple linear regression models, neural networks, and others, such as ensemble learning methods, hybrid models, and deep-learning approaches.

The outdoor environment significantly impacts the indoor environment, both thermally and in terms of air quality, as described in [33]. This interaction affects a building’s energy efficiency, comfort, and indoor air quality. This paper first reviews the coupling methods used to connect the outdoor and indoor environments. Then, it examines the impact of various outdoor physical elements, such as neighboring buildings, green spaces, road surfaces, water bodies, and the sky, on indoor thermal conditions. The research of [34] delved into the realm of wearable devices designed for environmental monitoring, investigating the progress in sensing technologies and their practical applications. The authors initiated their exploration by scrutinizing key pollutants, followed by an extensive examination of the sensing technologies utilized to gauge these parameters. Integrating personal activities and real-time environmental data facilitates correlating pollution levels with individual information like physical activity, location, and respiratory parameters, enabling a more accurate assessment of personal exposure to diverse pollutants. In all previous work, the absence of universally accepted indices for evaluating outdoor or indoor air quality still needs to be solved.

## 3. Materials and Methods

We now detail the proposed architecture for the monitoring system. The architecture is detailed at the level of the general organization of the components within the system (Section 3.1), at the level of the individual architecture of the elements (Section 3.2), and at the level of innovative components (Section 3.3).

### 3.1. The Monitoring Architecture

**Figure 1:**
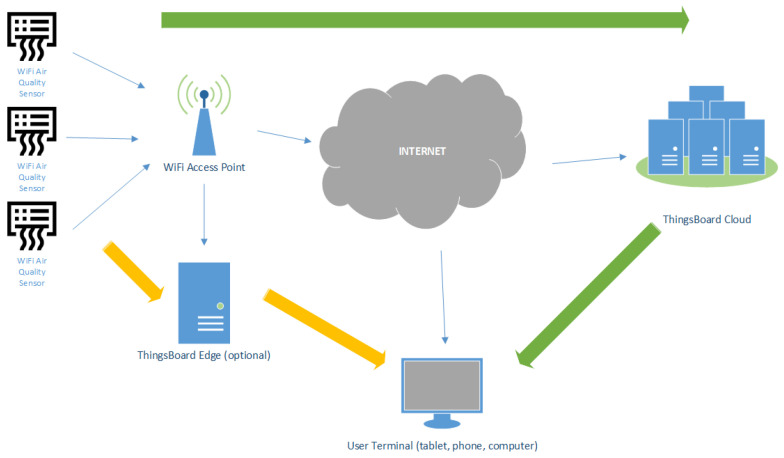
The architecture of the monitoring system.

The components of the system are the air quality monitoring devices, the ThingsBoard cloud service for storing and processing data, and the user terminal for viewing the information. Air quality monitoring devices have WiFi network communication capabilities and can connect to a local network infrastructure to send data to the ThingsBoard cloud service via the MQTT protocol. The data transmitted by the monitoring sensors are stored and processed by the cloud service to form an efficient visualization for the user. The information route is presented in Figure 1 through the green arrows.

The user’s terminal, the actual visualization of the data from the monitored home, can be anywhere. If the monitored home does not have a stable or high-speed Internet connection, an additional edge component (ThingsBoard edge) can be used. In this case, the monitoring devices will report the information to the edge device through the local network, which will then store the data locally and display the local viewing interface based on them without needing an Internet connection. The edge component does not aim to replace the cloud service, being a device with limited resources. The edge device synchronizes the data with the cloud service for long-term storage and analysis at regular intervals and when the Internet connection is available. The edge device works as a buffer for the data sent by the monitoring devices, allowing short-term local monitoring without an Internet connection. The flow of information in this case is represented in Figure 1 by the orange arrows.

### 3.2. Components and Methods for the Monitoring System

#### 3.2.1. IAQ Monitoring Device

The research team designed and made the IAQ monitoring device specifically for this study. The low-cost device is based on an easy-to-install solution that can be easily deployed on a large scale. The parameters are monitored using a high-integration digital sensor, Sensirion SEN55 [35]. Launched in April 2022, this sensor has a low price but high performance in sensing capabilities. It is already used in commercial indoor air quality monitoring equipment, which was recently found on the market from well-known companies [36]. As seen from Table 2, Sensirion SEN55 is a sensor capable of measuring seven environmental parameters (temperature, humidity, VOC and NO_x_ index, concentrations of PM1, PM2.5, PM4, and PM10) with a lifespan of over ten years and integrating several sensors’ functionalities into one. This leads to a decrease in the cost and complexity of the final monitoring device.

The practical, functional part of the monitoring device is based on a NodeMCU development board equipped with an Espressif ESP8266 SoC microprocessor [38]. As in the case of choosing the sensor, the development board was selected with a low cost and complexity in mind to implement an easy-to-use solution. The total cost of a monitoring sensor (NodeMCU development board, SEN55 sensor, interconnection cable, case, power supply) reaches EUR 25 at the retail price level (not large series). For the price, the device comes with no screen display (it was designed to cost as little as possible), but we consider it to be ideal for being installed in multiple locations in the same home.

**Figure 2:**
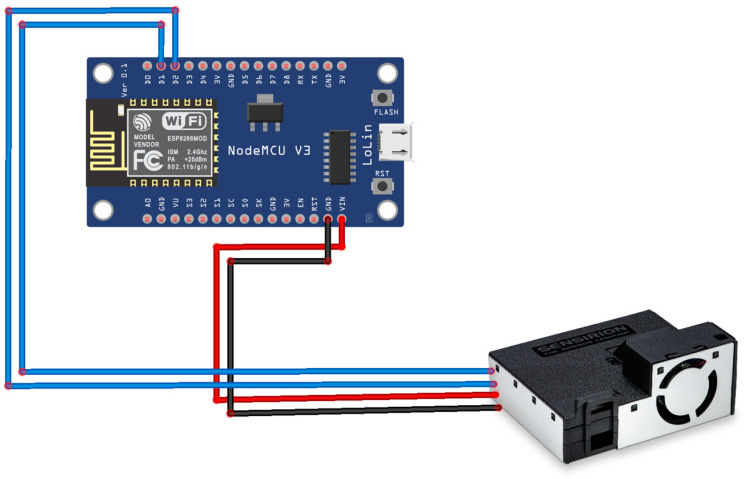
The interconnection between the SEN55 sensor and the NodeMCU development board (electrical diagram of the monitoring device).

The development board and the sensor are connected through the I2C bus, as shown in Figure 2. The device is powered by a regular mains power supply (a mobile phone charger) of 5 V, minimum 1 A.

The monitoring device can connect to a WiFi 802.11 b/g/n 2.4 GHz network with WPA/WPA2 security. The program running on the monitoring device was written in Arduino IDE 1.8.19 with the ESP8266 Community 3.0.2 extension installed and the following libraries: Sensirion I2C SEN5x 0.3.0, ArduinoJson 6.19.4, PubSubClient 2.8.0, Seed_Arduino_mbedtls 3.0.1, TBPubSubClient 2.9.1, and ThingsBoard 0.9.5.

**Figure 3:**
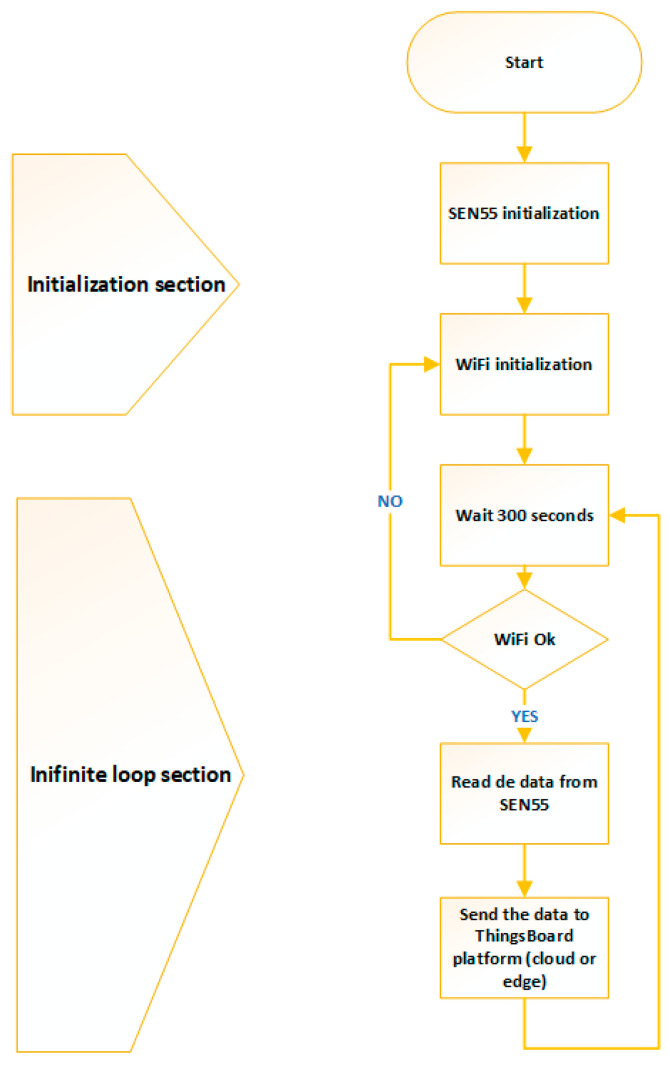
Functional diagram of the monitoring device.

The monitoring device program contains two sections: an initialization section and a section executed in the infinite loop (the operation diagram is presented in Figure 3). Initialization for all components includes setting up the I2C connection, initializing the SEN55 sensor, and establishing the network connection. The main section running in the infinite loop has a delay of 5 min (300 s), after which it reads the data from the SEN55 sensor and sends them to the ThingsBoard platform (edge or cloud version, from the point of view of the monitoring device there is no difference). At each cycle, the WiFi connection is checked, and if there is a problem, its initialization is resumed.

#### 3.2.2. Clouds Services

In the case of our IAQ monitoring system, the monitoring devices connect via the Internet to the ThingsBoard platform. The ThingsBoard platform [39] is an open-source IoT platform that allows the development of highly complex IoT projects. The functionalities of the ThingsBoard platform include (but are not limited to) the easy management of IoT devices connected to the platform; secure authentication key for each device; working with various IoT protocols for connecting IoT devices (HTTP, MQTT, CoAP, LwM2M); featuring data storage (in SQL and NoSQL databases) and data processing; the implementation of dashboards for advanced data visualization; user management for differentiated access for device management or just for viewing data; and allowing the interconnection of data visualization applications with external services.

The ThingsBoard platform is scalable, allowing the implementation of projects from the smallest to massive projects. The ThingsBoard platform can be used as a cloud instance hosted by the platform developer company or it can run on a dedicated server (or virtual machine) or on several servers with different functionalities (database, message queue, user interface) to ensure greater computing power in the case of large projects.

For the practical validation of the system carried out in this paper, a cloud instance running ThingsBoard v3.6.2PAAS was used on the ThingsBoard cloud. The size of the test performed and the running costs (the cloud instance starts at USD 10 per month at the time of this writing) determine how to use the platform.

**Figure 4:**
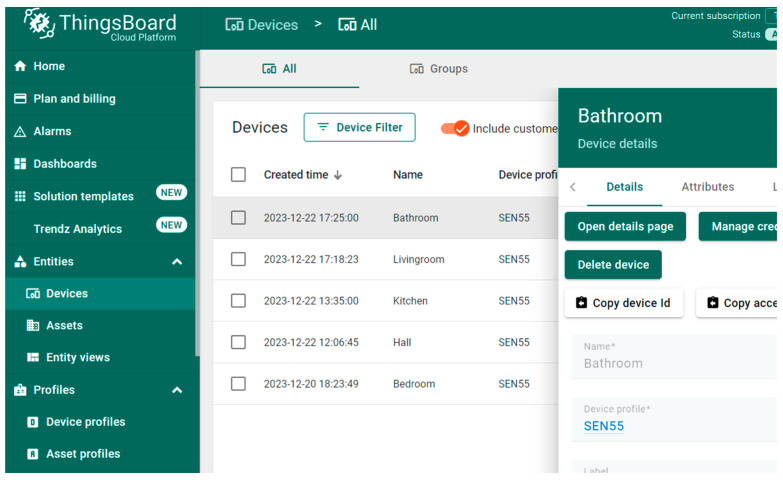
The management interface of connected devices—ThingsBoard cloud platform.

In addition to the management functionalities of the connected monitoring devices (Figure 4) and recording the data sent by the devices, the ThingsBoard platform also fulfills the following functionalities within the proposed architecture:

- Calculating interval averages for the parameters whose evaluation is performed this way.
- Receiving outdoor air quality data from an external specialized service via the Internet.
- Forecasting in advance of the evolution of monitoring parameters (described in Section 3.4).
- Building simplified visualization for the user (described in Section 3.2.3).

**Figure 5:**
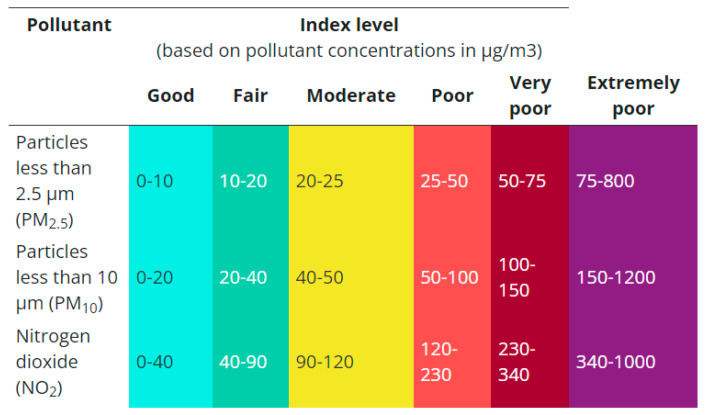
Outdoor index level for 24-h averages for PM2.5 and PM10 concentrations (source: European air quality index [40]).

The average calculation per interval is made for the concentration values of PM2.5 and PM10, whose accessibility thresholds are made according to the average per hour and per 24 h. Figure 5 shows the index levels of the two parameters for 24-h standards according to the European Environment Agency. At the level of the ThingsBoard platform, a rule-chain is defined, which, for each received value (once every 5 min), calculates the two averages (per hour and 24 h) for the PM2.5 and PM10 parameters. The estimated average values are saved in the database together with the data from the monitoring devices. The two values are dynamic; they represent the last hour and the previous 24 h every 5 min, respectively. These values are not calculated once an hour or once a day.

**Figure 6:**
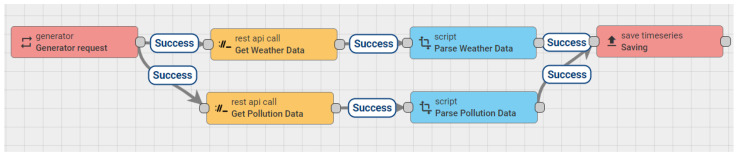
Rule-chain within the ThingsBoard platform that automatically brings environmental parameters using the OpenWeather service.

The monitoring system proposes the simultaneous use of data from indoor air quality sensors and data with environmental information from the outside. External environmental data are automatically retrieved by the ThingsBoard platform from the free external service OpenWeather [41]. The data are brought at 5-min intervals and saved in the database with the data from the monitoring devices through a rule-chain (Figure 6) defined at the platform level. The retrieved data contain information about external temperature, humidity, and pollution (PM2.5, PM10, SO_2_, NO_2_, NO, CO, O_3_, NH_3_, and the air quality index AQI).

The ThingsBoard cloud platform plays a vital role in the proposed architecture, implementing both basic functionalities related to device management and information recording from the devices and functionalities for processing and completing the information necessary to provide the user with a valuable vision of indoor air quality. Another primary functionality of the ThingsBoard platform is displaying data in an accessible format for the user, which will be presented next.

#### 3.2.3. User Interface

**Figure 7:**
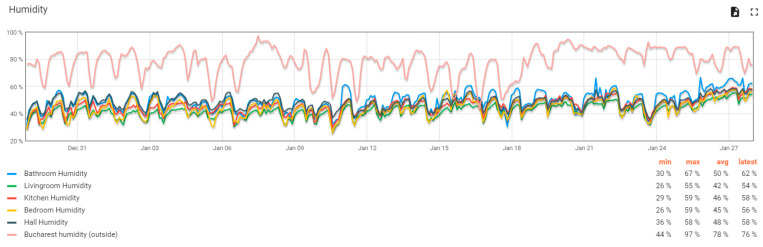
Example of an evolution graph for the humidity parameter for several indoor monitoring devices for a 30-day interval.

The user interface offered by the ThingsBoard platform is based on dashboards in which the information recorded in various forms of organization can be displayed. Within the monitoring system, two dashboards were designed for the user to visualize and evaluate the indoor air quality situation. The first dashboard is a classic interface that allows viewing of the evolution of parameters over variable periods. Figure 7 illustrates one of the evolution graphs from this dashboard for the humidity parameter. All graphs display the maximum and minimum values, the average value, and the last value for the displayed parameter. These graphs have an advanced analysis character and allow accurate diagnosis of the evolution of a specific parameter. This type of dashboard is usually used by advanced users who want to understand a particular situation of developing one or more air quality parameters.

**Figure 8:**
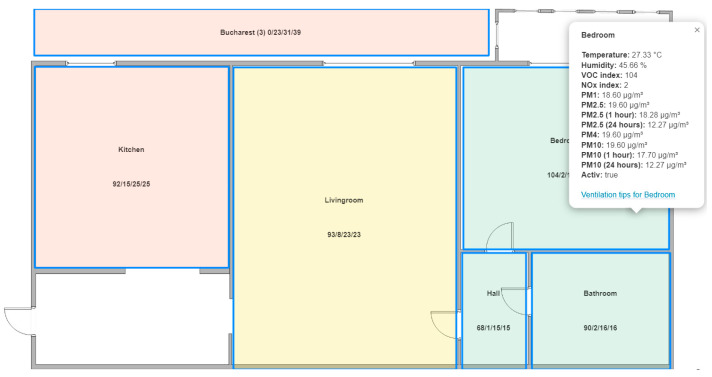
Dashboard for spatial visualization of indoor air quality through colors.

The second dashboard designed for the user interface of the air quality monitoring system is based on a visual representation of the monitored space. It displays the last measured values for the previous parameters and a color representation of the air quality index. The dashboard allows the requesting of detailed numerical information about a particular area (as seen in Figure 8).

The dashboard for spatial visualization uses a color code inspired by the European air quality index [40]. For the exterior (the rectangle at the top outside the apartment area—Bucharest), the exact color codes (Figure 5) and the values of the polluting agents PM2.5, PM10, and NO_2_ are used. Several pollution levels (good, fair, moderate, poor, very poor, extremely poor) and the associated representation colors are kept for the interior. Still, instead of using the average values for 24 h, the average values for 1 h are used for the polluting agents PM2.5 and PM10. This allows a much better dynamic of reporting to the user and generates a much tougher air quality assessment system. The proposed monitoring system aims to inform the user as quickly as possible about a possible problem related to air quality. The average of the values over the last 24 h is a valuable parameter for the global monitoring of the long-term evolution of air quality. Still, it is of little relevance for a specific moment. If the pollution limits are met for the average at the 1-h level, then they are certainly met at the 24-h average level. However, the 1-h average use is more severe than the 24-h average, leading to a stricter implementation. The national legislation (from Romania) does not specify limits for monitored polluting agents [42]. The European legislation is being revised (the old EU Directive 2008/50/EU is being revised), but the revision will likely be based on the latest version of the WHO guideline [8]. Considering that the WHO Global Air Quality Guidelines [9] apply to indoor and outdoor air [8] and specify stricter limits than the previous version from 2005, the approach proposed by the monitoring system in this paper is justified.

For the parameters VOC index and NO_x_ index, which are values without a unit of measure, the manufacturer’s evaluation information [43,44] was used. Table 3 presents the centralized way of establishing the indoor air quality index used in the spatial visualization dashboard.

**Figure 9:**
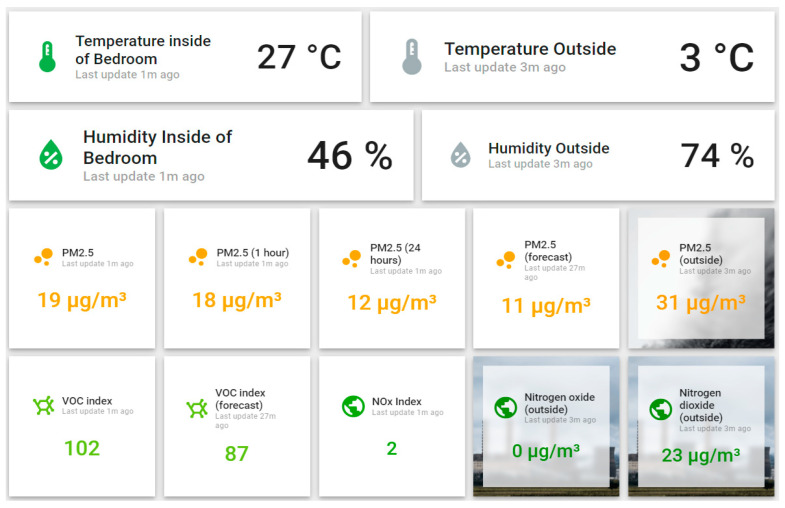
Visualization is oriented toward the comparative analysis of air quality parameters to make a ventilation decision.

Within the same dashboard, you can access an additional view that focuses on deciding related to the ventilation of a particular space. By accessing “Ventilation tips,” you navigate to an idea that contains information about a specific area (Figure 9). This view compares the temperature and humidity inside/outside and the pollution parameters. In this way, the user can make a correct decision regarding the opportunity to ventilate a specific space to obtain good indoor air quality and maintain the desired thermal comfort.

Through the two visualizations, the monitoring system’s user interface addresses the information needs of all users (from beginners to advanced). It facilitates the comparative analysis of indoor and outdoor environmental parameters to decide on the selective ventilation of the rooms in the house.

### 3.3. The Monitoring Scenario

**Figure 10:**
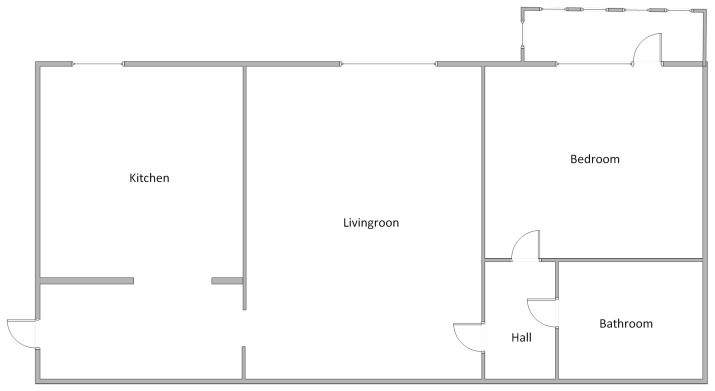
Layout of the test apartment.

A monitoring scenario was implemented to test and validate the utility of the proposed architecture. The test location was a small apartment in downtown Bucharest, Romania. The apartment has two rooms, a bathroom and kitchen, and an area of 40 square meters. The layout of the test apartment is presented in Figure 10. It is one of the most common types of apartments in Bucharest. Two adults and a small pet inhabit the apartment. During the testing period, life in the condo proceeded usually; the tenants followed their usual routine.

The apartment is not equipped with automatic ventilation systems of any kind. Ventilation is carried out exclusively manually through the windows present in each room of the apartment. The apartment is heated by hot water radiators connected to the municipal central heating system. Food is prepared using a natural gas stove and an electric oven in the kitchen. The apartment was completely renovated two years ago, and no new decorations or furniture have been introduced in the last six months.

Five monitoring devices were used to monitor the air quality in the kitchen, living room, bedroom, hall, and bathroom. The devices were positioned on walls not exposed to direct solar radiation at a height of 1.5 m from the floor. The monitoring devices worked continuously 24/7 and used the WiFi network infrastructure present in the apartment. The connection to the Internet was made through a high-speed optical fiber, so an edge ThingsBoard component was not used.

The user interface was made available through an Android tablet with a 12.7″ screen positioned in the living room and working 24/7. On the tablet screen, the continuous display of the colorful spatial representation of the apartment was configured. In this way, users could check the air quality in all the rooms in the apartment. They could see the distinct opportunity to ventilate each room depending on the indoor and outdoor pollution conditions.

### 3.4. Innovative Analysis Elements in Indoor Air Quality Monitoring

The monitoring system architecture and the test performed to validate the technical solution introduce the following innovative elements for indoor quality monitoring:

- The comparative presentation of indoor and outdoor air quality information so that the user can decide whether the ventilation at a given time leads to the improvement of the indoor air or not and, at the same time to be able to evaluate the impact of the ventilation action on the thermal comfort of their own home.
- Individual monitoring of the rooms in the house so that the user can see the source of air quality degradation and can intervene by ventilating the affected area. The presentation of data in a central console in a spatial format that transmits the information simply using colors allows the user to visually check the entire home easily and quickly (Figure 8).
- The room’s analysis interface presents, in addition to the data from inside and outside, the evolution forecast for the pollution parameters, the VOC index, and PM2.5 concentration (Figure 9). Every hour, the ThingsBoard platform runs a Prophet prediction algorithm [45] and provides the parameter’s value in one hour based on the data collected in the last 14 days. In this way, the user can decide to ventilate the room in a preventive manner to avoid a decrease in air quality.

## 4. Results and Discussion

As part of the testing process, an interval of 1 month (between 1 January 2024, at 0:00 a.m. and 1 February 2024, at 0:00 a.m.) was chosen to analyze the information the indoor air quality monitoring system recorded. The dataset included values sent by the five monitoring devices tested at 5-min intervals, 12 recordings per hour per device, 288 recordings per day per device, 8928 recordings in the analyzed interval per device, and 44,640 recordings. Each recording consisted of eight values representing temperature, humidity, VOC index, NO_x_ index, and concentrations of PM1.0, PM2.5, PM4.0, and PM10. The VOC and NO_x_ index values were retrieved and stored as integer values; the other values were retrieved and stored as float values. In addition to the data coming directly from the sensors, the platform calculated every 5 min the average values at the hourly and 24-h levels for the PM2.5 and PM10 concentration parameters. The PM2.5 and PM10 parameters are the only parameters related to particulate matter concentrations whose limits appear in the current air quality standards. For this reason, they are also the singular values for which the two averages were calculated and analyzed. Calculating these averages generated an additional dataset of 8928 records per day per device, 44,640 records in total (each paper composed of 4 float values).

The ThingsBoard platform brings data related to the weather and pollution level at the city level (Bucharest) from the OpenWeather platform once every 5 min and saves them in the database. The recording made with data from OpenWeather contains the outside temperature and humidity, atmospheric concentrations for PM2.5, PM10, CO, NO, NO_2_, O_._, NH_3_, and AQI (air quality index). The only integer value is AQI; all other values are float values. Considering the collection interval, during the analysis period of 31 days, 8928 records were collected about the quality of the air outside.

The last dataset analyzed comes from the indoor air quality forecasting algorithm. Every hour, the Prophet algorithm implemented at the ThingsBoard platform level forecasts the evolution values for the VOC index and the PM2.5 concentration for over an hour. The forecast set analyzed contains 24 values for each of the two parameters per day, 744 records per device, and 3720 values in total.

### 4.1. Recorded Data

The average temperature in the apartment during the test period was 25.28 °C with variations between 19.2 °C and 31.6 °C. The lowest temperature was recorded in the bathroom as an absolute value (19.2 °C) and an average (24.5 °C) for the entire period. The most significant temperature fluctuation (9 °C) was also recorded in the bathroom. The warmest room was the living room, both in terms of minimum (23.2 °C) and maximum (31.6 °C) and average values (26.6 °C). The most stable temperature was recorded in the hall with a variation of only 4 °C. The highest average humidity was recorded in the kitchen (54%) and the lowest (48%) in the living room and bedroom. The average humidity per apartment was 50.8%. The most significant variation of the humidity parameter was recorded in the bathroom (37%). Table 4 presents the complete data on the average evolution of ambient temperature and humidity during the test period.

From the point of view of pollution with volatile organic compounds and nitrogen oxides, as average values, the kitchen is the most polluted place in the apartment. However, it can be noted, according to Table 5, that dangerous maximums can also appear in the rest of the apartment, with a maximum VOC index of 496 in the bedroom and a complete NO_x_ index of 31 in the bathroom. In general, the analysis of the two indicators, VOC index and NO_x_ index, showed averages that fit the air in the apartment to maximum quality (VOC index < 150 and NO_x_ index < 40). This is because there are no indoor polluting factors for the two categories of pollutants. The only combustion source was a gas stove that was rarely used; cooking was performed using an electric oven. There were no artificial sources of volatile organic compounds, and all renovations and new furniture elements were introduced into the apartment more than six months before the test.

From the point of view of pollution with fine particles, the variations in PM2.5 and PM10 concentrations, instantaneous values (measured), and average values (calculated) at intervals of 1 h and 24 h were analyzed. As seen from Table 6, the most affected room for both categories of particles (PM2.5 and PM10) is the kitchen, both as maximum values and as average values for the entire test interval. The least “dusty” room is the bathroom, this being normal because the bathroom contains the least decorative textile elements that can generate such particles. The average values for the apartment (20 µg/m^3^ for PM2.5 and PM10) generally classify the air quality, from this point of view, as fair.

The kitchen is the only room with an average concentration of PM2.5, which places it at the “moderate” level from the point of view of air quality. This can be explained, as in the case of the VOC and NO_x_ indices, because cooking in the kitchen is one of the most important sources of pollution in a modern apartment. In the tested apartment, there is no way to mitigate these effects; the kitchen is not equipped with a kitchen hood and has no additional ventilation.

The testing period was in January, the winter month in Bucharest, Romania, characterized by low temperatures and high humidity. Pollution at the city level was relatively quiet, as seen in Table 7. The primary purpose of monitoring the outdoor parameters was to provide the user with extensive information on ventilating the apartment effectively. However, the collected data allow for further analysis. The correlation between indoor and outdoor values can provide crucial information on the pollution level in the city, the air quality inside the apartment, and, additionally, the influence of the cold winter weather on indoor thermal comfort. The values for which the correlation was tested were temperature, humidity, NO and NO_2_ concentrations (in correlation with the NO_x_ index), and PM2.5 and PM10 concentrations (in correlation with the hourly average indoor concentrations of PM2.5 and PM10).

### 4.2. Correlation of Indoor and Outdoor Air Quality

The correlation between the value series (internal and external) was assessed using the Pearson coefficient (Equation (1), where *x* and *y* are the two-time series for which the correlation is checked, with the $\bar{x}$ and $\bar{y}$ being the average values).

(1) $$r=\frac{\sum \left(x-\bar{x})(y-\bar{y}\right)}{\sqrt{\sum {\left(x-\bar{x}\right)}^{2}\sum {\left(y-\bar{y}\right)}^{2}}}$$

The correlation was analyzed on the last 15 days of the test period (17 January 2024–31 January 2024) using hourly averages for all time-series values. From Table 8, you can see the correlation between the rooms of the apartment and the correlation with the external values in the case of air temperature and humidity. The internal/external correlation in the case of temperature is weak, varying between 0.36 and 0.53. This indicates the excellent quality of the thermal insulation of the apartment and the correct operation of the indoor heating installation. In the case of humidity, there is no correlation between the internal and external values (the Pearson coefficient is negative). This can be explained by the substantial differences between the internal and external temperatures of almost 23 °C. Strong correlations can be observed, both in the case of temperature and humidity but also in the case of polluting agents (Table 9 and Table 10), between the rooms that form an open space without doors (kitchen and living room).

In the case of the correlation of polluting agents (PM2.5 and PM10—Table 7, NO and NO_2_—Table 8), a weak (PM2.5 and PM10) and very weak (NO and NO_2_) correlation between interior and exterior can be observed. Considering the average pollution values (presented in Table 5 and Table 6), which indicate a higher level of pollution indoors than outdoors, it can be concluded that indoor pollution is not caused by external pollution but primarily by internal sources (human activities—cooking, cleaning, other activities). It is true that external pollution can contribute to the increase in internal pollution, but not in a directly proportional manner, but rather as a cumulative factor.

The correlation between the indoor NO_x_ index and the outdoor concentrations of NO and NO_2_ (Table 9) indicates that, even though it is a polluting agent without a significant impact on air quality in the case of the tests performed, there is no correlation between indoors and outside. Moreover, the correlation between the rooms of the same apartment is less intense than in the case of PM2.5 and PM10 concentrations. This means this pollution factor has a spatially limited effect, generated by internal local sources that only affect a small area.

### 4.3. One Step Ahead of Indoor Air Pollution

One of the innovative elements proposed by the monitoring system in this paper is the forecasting component of indoor pollution indicators. The forecasting component was used for the VOC index and PM2.5 concentration indicators using an internal mechanism of the ThingsBoard platform that allows estimation of the value of a parameter in advance using a series of previous values. Three forecasting mechanisms provided by the ThingsBoard platform [46], Fourier transformation, linear regression, and Prophet were evaluated. These mechanisms allowed real-time forecasting of the evolution of the two pollution parameters, but, like any ready-to-use tool, they did not allow the refinement of the forecasting process at the level of the parameters of the algorithms. The only parameter configurable at the platform level is the size of the set of values underlying the forecast. Several successive tests have shown an approximately satisfactory behavior for a time series with hourly average values for 14 days as the basis for forecasting the value over one hour.

**Figure 11:**
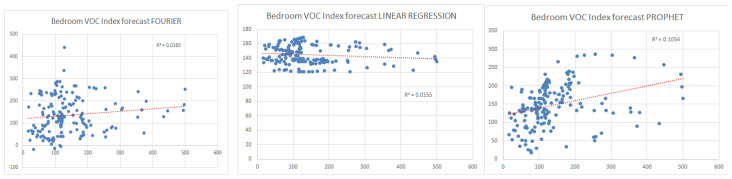
Comparison of prediction performance for VOC index in the bedroom for the Fourier transformation, linear regression, and Prophet algorithms (trendline with red).

The three forecasting methods were compared by calculating the R^2^ coefficient (the square of the Pearson correlation coefficient) for the series of measured and forecasted values. As can be seen from Figure 11, where a comparison was made between the forecasts obtained using the three methods for the VOC index for the bedroom, the least wrong was the Prophet method with an R^2^ equal to 0.1054 compared to 0.0165 for Fourier transformation and 0.0155 for linear regression. Better results obtained by the Prophet forecasting method can be justified by the seasonal patterns component integrated into the algorithm. The poor forecast obtained, in general, is justified by the chaotic nature of the parameters and the unrefined use of the forecasting algorithms.

The Prophet algorithm is based on an additive regression model with a linear growth curve trend. The advantage over other forecasting algorithms is the seasonal component modeled using the Fourier series [47]. The superior results of the Prophet algorithm in the presented case can be explained by combining the methods to determine the repetitive events that are the only predictable component in the forecast of the evolution of the air quality at the beginning. In addition to completely random events that lead to unpredictable indoor air pollution, a series of events relate to the daily/weekly/seasonal routine of people living in the monitored premises.

The purpose of implementing the prediction mechanism was to demonstrate the possibility of integrating a real-time forecasting mechanism of pollution parameters within an indoor air quality monitoring system. Obtaining a quality forecast that comes as close as possible to the natural variation in the parameters is a challenge and represents an additional research topic.

**Figure 12:**
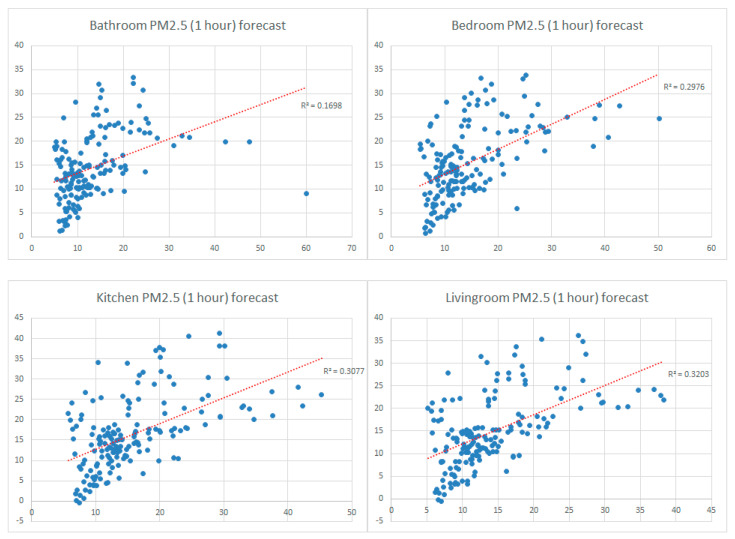
Analysis of the PM2.5 parameter forecast quality using the Prophet forecasting method (trendline with red).

Figure 12 shows the forecast behavior (based on the R^2^ coefficient) for the variation in the hourly average concentration of PM2.5 in different rooms in the apartment using the Prophet method. The quality of the forecast for the PM2.5 variation is superior (R^2^ is between 0.1698 and 0.3203) compared to the VOC index forecast, which means that the PM concentration has a more predictable character generated by human activities that are repeated according to a specific schedule (daily cleaning, arranging things in the house, the period of absence as a result of the daily activity at the office, etc.). The link between the variations in indoor pollution parameters and people’s daily routines is an exciting direction of future analysis that can be researched with the help of the monitoring system proposed in this paper.

### 4.4. Added Value for the User

The monitoring architecture proposed in this paper brings more benefits to the user, allowing a complete and detailed analysis of the evolution of the air quality in their apartment. The comparative study between inside and outside allows the user to make efficient ventilation decisions considering the thermal comfort inside and the polluting factors outside. Forecasting the evolution of pollution parameters allows venting choices to be made to prevent periods of pollution that repeat regularly based on patterns recorded in the past. For advanced users who want to better understand the activities that lead to indoor air pollution, the user interface provided by the monitoring system can be transformed into a “forensic” investigative tool.

**Figure 13:**
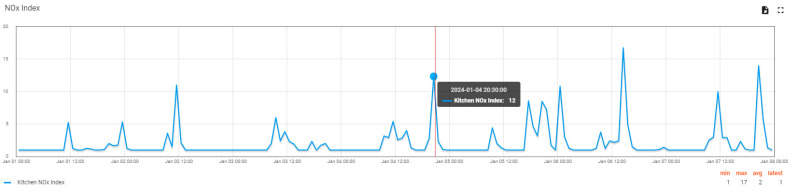
NO_x_ index variation graph in the kitchen over seven days (data can be saved from the user interface using the icons on the top right).

A visual analysis of the evolution graph of the NO_x_ index in the kitchen over seven days (Figure 13) can lead to the observation that the significant increases in the parameter follow a simple rule: it happens around lunchtime (12:30–14:30) and in the evening (20:30–21:30). Both periods of the day coincide with the use of the gas stove for heating or food preparation. In this way, it is straightforward to understand the connection between human activity and the increase in pollution in the apartment.

**Figure 14:**
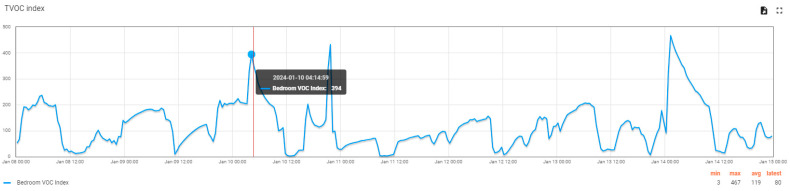
VOC Index variation graph in the bedroom over seven days (data can be saved from the user interface using the icons on the top right).

The VOC index in the bedroom (Figure 14) shows periods of growth, even dangerous in terms of pollution level, during the night (between 00:00 and 08:00). This is explained by the fact that during this period two adults sleep in this room. No ventilation operations are performed during this time. Human breathing is an essential source of VOCs, and the gases produced by the room occupants accumulate during the night. The increased VOC levels observed (maximum index 467) may suggest too little room air volume (either from the room’s physical dimensions or the occupation of the space in the room with objects).

**Figure 15:**
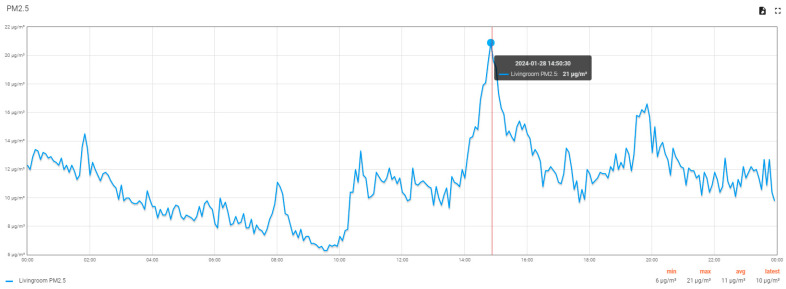
The graph of the variation in the concentration of PM2.5 in the living room on Sunday, 28 January 2024 (data can be saved from the user interface using the icons on the top right).

Let us analyze how the concentration of PM2.5 varies in the living room on a Sunday (Figure 15) when both apartment tenants are at home. People’s activity can be deduced from the variation in PM2.5: the period up to 08:00 is a period of inactivity. At 08:00, a tenant wakes up, and at 10:00, domestic activities begin and end at 2:00 p.m. There is a siesta period followed by evening domestic activities until 08:00 p.m., the evening rest period, and the cycle resumes.

The proposed monitoring system addresses the needs of a wide range of users, leading to the quick decision to plan a home’s ventilation, helping to evaluate indoor pollution sources and understand the impact of personal activity on indoor air quality.

To correctly evaluate the functionality of the user interface implemented in the monitoring system, the opinion of several users was requested through an online questionnaire. All users had online access to the user interface of the monitoring system to be able to express their opinion, knowing what it is about. In total, 33 people expressed their opinion about the user interface of the monitoring system presented in this paper; 20 people were from the over 45 age category, 10 people from the 34–45 age category, and 3 people from the 25–35-year age category; 21 respondents declared that they have basic knowledge about IAQ monitoring, 7 that they know nothing about this, 4 that they are familiar with IAQ monitoring systems, and 1 respondent that he is a specialist in the field. The evaluation questionnaire contained 10 questions. In total, 7 questions were of the Yes/No type and mainly evaluated the opinion of the users on the original functionalities of the proposed monitoring system (5 out of 7 questions), the distribution of the answers (Table 11) showing with a large majority a positive opinion about these functionalities. Another question from the questionnaire was “What role does price play in the purchase of an indoor air quality monitoring system?”. This question tried to evaluate the importance of low-cost IAQ monitoring systems in everyday life. A total of 29 out of 33 respondents stated that a low price is decisive in buying such a system, 1 person that the high price is a reason for purchase, and 3 people believed that the price has nothing to do with the purchase decision.

To the question “Which environmental and air quality parameters do you think need to be monitored in a home?”, the people answered that it is necessary to monitor dust (33 respondents), humidity (32 respondents), temperature (29), VOC (18), NO_x_ (18), and other parameters (1). The IAQ monitoring system implemented and presented in this paper fully satisfies the wishes expressed by the people surveyed. The last question requested the general opinion on the user interference of the IAQ monitoring system and was a free-answer type. The answers were as follows: “Very good”, “Very interesting idea. I am interested in buying”, “Extremely intuitive and pleasant in style”, “An important system that can influence the quality of life”, “Good”, “It is easy to understand and follow. The visual representation is intuitive. The color coding is logical and makes sense. It is easy to navigate between the desired rooms”, “It is very easy to follow”, “Clear and concise”, “The user has something to gain”, “It is a modern interface”, “Useful”, “Good idea, but the price of the system should be affordable for everyone”, “Ok”.

## 5. Conclusions

Indoor air quality is a significant problem but should be more well-known by the public. The connection between the low quality of indoor air and related health problems, the long time spent inside buildings, and the influence of household activities on the air quality in homes are information that must reach all people. The monitoring system proposed in this paper comes to the aid of users and provides them with a tool of variable complexity depending on the need and technical training.

The monitoring system is a low-cost solution that is easy to implement, maintain, and use. The user interface designed within the system is organized so that the user benefits from help in the current activities of manual ventilation of the house as well as in the detailed analysis of the indoor air quality and the sources of pollution that decrease the air quality.

More and more people are joining urban agglomerations and living in block apartments with modest dimensions. The utility of indoor air monitoring systems is essential in ensuring decent air quality and tenant health in personal flats. The COVID-19 pandemic has shown that there are situations where the private home is the place that needs to be taken care of the most.

The proposed monitoring system is addressed to a well-defined segment of users or, rather, to a category of living spaces that do not have automatic ventilation systems, and where there is no possibility of installing them. This problem also exists in closed spaces other than personal homes, such as educational spaces or office buildings that operate in old buildings (some even national heritage). The scalability of the cloud system allows the use of the monitoring system in buildings with many premises (rooms). It is not the purpose of the monitoring system to interface with automatic ventilation systems (HVAC) or centralized management systems (BMS). However, the ThingsBoard cloud system allows interconnection with other informational systems through various protocols. This extremely flexible connectivity can be used in transition scenarios for limited periods in which such systems are installed. The monitoring devices proposed within the monitoring architecture in this paper can be an example of cost reduction for monitoring systems and automatic ventilation.

The development of IoT systems currently involves highly complex architectures that benefit from information from other systems as well as automatic learning techniques. The architecture proposed in this paper is part of the current development trend of IoT systems, offering both a processing part closer to the monitored system (edge computing) and informational collaboration with other systems (retrieving information about pollution from the outside), as well as advanced forecasting techniques based on the additive regression model Prophet.

The proposed architecture, tested in a naturally relevant environment, is perfectly functional and provides simple and helpful use for end-users. The method used to validate the original elements of the proposed monitoring system was based on the evaluation of the satisfaction of the system users. The conclusion of the technical tests and the evaluation of the users’ responses show that the original elements of the monitoring system achieve their proposed goal of improving the end-user’s experience. More than that, the apartment tenants where the system was tested and users who have used the system for an extended period expressed their firm desire to keep the monitoring system for long-term use.

## Figures and Tables

**Table 1:** State-of-the-art existing IAQ monitoring systems.

IAQ Monitoring System Described in	Ventilation System	Multi-Zone Technology	Edge Computing Capabilities	Monitored Parameters	Index Report	Exterior Data	Real-Time Forecasting	User Interface	Cost of Monitoring Device
This paper	Manual	WiFi, IoT	Yes	Temperature, humidity, PM1.0, PM2.5, PM4.0, PM10, VOC index, NO_x_ index	Color-Based	Yes	Yes	Charts; Multi-zone spatial representation; Cards; Tabular; Numerical	Custom, EUR 25
[21]	Natural ventilation	WiFi, IoT	No	PM1.0, PM2.5, PM10	No	No	No	Graph representation; Tabular	Low-cost system
[22]	NS	IoT Cloud, Zigbee, WiFi	Yes	Multiples gases and particulate matter, humidity, temperature	Yes	No	No	Tabular	Low-cost system
[23]	Central AC system	IoT, WSN Smart mobile, ethernet, WiFi	No	CO_2_, CO, SO_2_, NO_2_, O_3_, Cl_2_, temperature, relative humidity	No	No	No	Chart representation	NS
[24]	Natural ventilation	WiFi, cellular networks, IoT	No	PM, temperature, humidity, formaldehyde	No	No	No	Numerical; Chart representation	Cost-effective, USD 96.4
[2]	Natural and ventilation System	IoT, distributed ledger technology	No	PM2.5, PM10, HCHO, TVOC, C_6_H_6_, CO_2_, CO, O_3_, NO_2_, temperature, humidity, illumination, noise	No	No	No	Chart representation	Low-cost system
[25]	NS	WSN, ZigBee, cloud computing	No	VOC: benzene, toluene, ethylbenzene, and xylene	No	No	No	Chart representation	Low-cost system
[26]	Ventilation system	Wireless IQRF	No	Carbon dioxide, temperature, relative humidity, atmospheric pressure	No	No	No	Graph representation	NS
[27]	HVAC system	IoT	No	Temperature, relative humidity, CO_2_, CO, particulate matter (PM2.5 and PM10)	Yes	No	Yes	Graph representation	NS
[28]	HVAC system	IoT, machine learning, WiFi, mobile network, Mobile app	No	CO_2_, PM 2.5, NO_2_, CO, methane, humidity, temperature	Yes	No	Yes	Graph representation	Low-cost system
[29]	Manual ventilation	IoT, Zigbee, WSN	No	PM2.5, CO_2_, temperature humidity	No	Yes	No	Tabular; Graph representation	Low-cost, USD 47.2
[30]	Central AC system	IOT, LoRa	No	CO_2_, PM2.5, PM10, TVOC, HCHO, ambient temperature, relative humidity	No	Yes	No	Tabular; Graph representation	NS

AC—air conditioning; NS—not specified.

**Table 2:** Sensirion SEN55 functional characteristics (extracted from [37]).

Parameter	Value
Mass concentration specified range (PM1, PM2.5, PM4, PM10)	0 to 1.000 μg/m^3^
VOC and NO_x_ index	1 to 500 index points
Temperature and humidity	−10 to 50 °C, 0–90% RH
On-chip humidity compensation	Yes
Compensated outputs	Temperature and relative humidity
Supply voltage	5 V
Peak supply current	100 mA
Interfaces	I^2^C
Lifetime	>10 years
Sensor startup time	50 ms
Sampling interval	1 s

**Table 3:** Custom indoor level index based on EAQI and WHO recommendations.

Polluant	Index Level					
	Good	Fair	Moderate	Poor	Very Poor	Extremely Poor
PM2.5 (1 h)	0–10 µg/m^3^	10–20 µg/m^3^	20–25 µg/m^3^	25–50 µg/m^3^	50–75 µg/m^3^	>75 µg/m^3^
PM10 (1 h)	0–20 µg/m^3^	20–40 µg/m^3^	40–50 µg/m^3^	50–100 µg/m^3^	100–150 µg/m^3^	>150 µg/m^3^
VOC Index	<150		<250	<400		<500
NOx Index	<20		<150	<300		<500

**Table 4:** Analysis of ambient temperature and humidity values for the five monitoring rooms during the test period (blue—minimum value, red—maximum value).

	Temperature (°C)				Humidity (%)			
	Min	Max	Var. Range	Average	Min	Max	Var. Range	Average
Bathroom	19.2	28.2	9	24.5	29	66	37	52
Livingroom	23.2	31.6	8.4	26.6	27	57	30	48
Kitchen	21.6	28.3	6.7	24.6	31	61	30	54
Bedroom	22.4	30.7	8.3	25.7	26	60	34	48
Hall	23	27	4	25	36	60	24	52
Average	21.88	29.16	7.28	25.28	29.8	60.8	31	50.8

**Table 5:** Analysis of pollution index values (VOC and NOx) for the five monitoring rooms during the test period (blue—minimum value, red—maximum value).

	VOC Index				NOx Index			
	Min	Max	Var. Range	Average	Min	Max	Var. Range	Average
Bathroom	7	494	487	55	1	31	30	2
Livingroom	4	479	475	121	1	19	18	2
Kitchen	7	466	459	133	1	23	22	3
Hall	13	492	479	70	1	26	25	2
Bedroom	4	496	492	100	1	18	17	1
Average	7	485.4	478.4	95.8	1	23.4	22.4	2

**Table 6:** Analysis of particulate matter concentrations (PM2.5 and PM10) for the five monitoring rooms during the test period (instantaneous values, 1-h average, and 24-h average, blue—minimum value, red—maximum value).

	PM2.5 (µg/m^3^)				PM10 (µg/m^3^)			
	Min	Max	Var. range	Average	Min	Max	Var. range	Average
Bathroom	2	243	241	18	2	244	242	19
Bedroom	3	263	260	20	3	264	261	21
Hall	2	272	270	17	2	275	273	17
Kitchen	3	475	472	24	3	481	478	24
Livingroom	3	319	316	21	3	323	320	21
Average	3	314	312	20	3	317	315	20
	PM2.5 (1 h) (µg/m^3^)				PM10 (1 h) (µg/m^3^)			
	Min	Max	Var. range	Average	Min	Max	Var. range	Average
Bathroom	2	193	191	18	2	193	191	19
Bedroom	4	210	206	20	4	210	206	21
Hall	3	244	241	17	3	246	243	17
Kitchen	4	393	389	24	4	401	397	24
Livingroom	4	258	254	21	4	262	258	21
Average	3	260	256	20	3	262	259	20
	PM2.5 (24 h) (µg/m^3^)				PM10 (24 h) (µg/m^3^)			
	Min	Max	Var. range	Average	Min	Max	Var. range	Average
Bathroom	7	65	58	19	7	65	58	19
Bedroom	9	78	69	21	9	78	69	21
Hall	6	67	61	17	6	67	61	17
Kitchen	10	101	91	24	10	101	91	24
Livingroom	8	83	75	21	8	83	75	21
Average	8	79	71	20	8	79	71	20

**Table 7:** Statistical data regarding outdoor temperature and humidity and NO, NO_2_, PM2.5, and PM10 concentrations outside.

	Temperature (°C)				Humidity (%)			
	Min	Max	Var. range	Average	Min	Max	Var. range	Average
Bucharest	−7	15	22	2	45	96	51	78
	NO (µg/m^3^)				NO_2_ (µg/m^3^)			
	Min	Max	Var. range	Average	Min	Max	Var. range	Average
Bucharest	0	22	22	1	2	58	56	13
	PM2.5 (µg/m^3^)				PM10 (µg/m^3^)			
	Min	Max	Var. range	Average	Min	Max	Var. range	Average
Bucharest	1	53	52	14	1	68	67	18

**Table 8:** The Pearson correlation coefficient over 15 days for temperature and humidity (green—strong correlation, yellow—no correlation).

Temperature						
	Bathroom	Livingroom	Kitchen	Hall	Bedroom	Bucharest
Bathroom	1	0.6886	0.6599	0.7807	0.6761	0.3751
Livingroom	0.6886	1	0.9554	0.8676	0.9019	0.5197
Kitchen	0.6599	0.9554	1	0.8409	0.8574	0.5354
Hall	0.7807	0.8676	0.8409	1	0.8303	0.3673
Bedroom	0.6761	0.9019	0.8574	0.8303	1	0.5041
Bucharest	0.3751	0.5197	0.5354	0.3673	0.5041	1
Sample Size:	360	360	360	360	360	360
Humidity						
	Bathroom	Livingroom	Kitchen	Hall	Bedroom	Bucharest
Bathroom	1	0.88	0.8336	0.9299	0.91	0.0096
Livingroom	0.88	1	0.9756	0.9514	0.9196	−0.0402
Kitchen	0.8386	0.9756	1	0.9331	0.8697	−0.092
Hall	0.9299	0.9514	0.9331	1	0.923	−0.0948
Bedroom	0.91	0.9196	0.8697	0.923	1	0.0817
Bucharest	0.0096	−0.0402	−0.092	−0.0948	0.0817	1
Sample Size:	360	360	360	360	360	360

**Table 9:** The Pearson correlation coefficient over 15 days for PM2.5 and PM10 concentrations (green—strong correlation, yellow—no correlation).

PM2						
	Bathroom	Livingroom	Kitchen	Bedroom	Hall	Bucharest
Bathroom	1	0.6749	0.6418	0.6867	0.751	0.3117
Livingroom	0.6749	1	0.987	0.8596	0.9595	0.2428
Kitchen	0.6418	0.987	1	0.8257	0.9403	0.2147
Bedroom	0.6867	0.8596	0.8257	1	0.8973	0.2668
Hall	0.751	0.9595	0.9403	0.8973	1	0.2856
Bucharest	0.3117	0.2428	0.2147	0.2668	0.2856	1
Sample Size:	360	360	360	360	360	360
PM10						
	Bathroom	Livingroom	Kitchen	Hall	Bedroom	Bucharest
Bathroom	1	0.6283	0.5938	0.7015	0.6384	0.2869
Livingroom	0.6283	1	0.9857	0.9587	0.848	0.2285
Kitchen	0.5938	0.9857	1	0.9394	0.8132	0.2033
Hall	0.7015	0.9587	0.9394	1	0.8842	0.2604
Bedroom	0.6384	0.848	0.8132	0.8842	1	0.2394
Bucharest	0.2869	0.2285	0.2033	0.2604	0.2394	1
Sample Size:	360	360	360	360	360	360

**Table 10:** The Pearson correlation coefficient over 15 days between the indoor NO_x_ index and the outdoor NO and NO_2_ concentrations (green—strong correlation, yellow—no correlation).

NOX/NO						
	Bathroom	Livingroom	Kitchen	Hall	Bedroom	Bucharest
Bathroom	1	0.7598	0.4558	0.9626	0.9111	0.0769
Livingroom	0.7598	1	0.8365	0.796	0.8684	0.0808
Kitchen	0.4558	0.8365	1	0.541	0.5921	0.051
Hall	0.9626	0.796	0.541	1	0.9361	0.1093
Bedroom	0.9111	0.8664	0.5921	0.9361	1	0.1219
Bucharest	0.0769	0.0808	0.051	0.1093	0.1219	1
Sample Size:	360	360	360	360	360	360
NOX/NO2						
	Bathroom	Livingroom	Kitchen	Hall	Bedroom	Bucharest
Bathroom	1	0.7598	0.4558	0.9626	0.9111	−0.068
Livingroom	0.7598	1	0.8365	0.796	0.8684	−0.0212
Kitchen	0.4558	0.8365	1	0.541	0.5921	−0.0196
Hall	0.9626	0.796	0.541	1	0.9361	−0.064
Bedroom	0.9111	0.8684	0.5921	0.9361	1	−0.0316
Bucharest	−0.068	−0.0212	−0.0196	−0.064	−0.0316	1
Sample Size:	360	360	360	360	360	360

**Table 11:** Answers to the questionnaire about the user interface of the IAQ monitoring system.

Question	Yes	No
Do you consider it necessary to use an indoor air quality monitoring system?	32	1
Do you currently use an air quality monitoring system in your own home?	3	30
Do you think it is necessary to monitor the air quality individually in each room for your own home?	28	5
Do you consider that the visual spatial representation of the monitored home improves the user experience of the air quality monitoring system?	29	4
Do you think that an indoor air quality monitoring system should also present the outdoor air quality to facilitate the decision to ventilate the space?	31	2
Do you think that using colors to indicate indoor air quality improves the user experience?	33	0
Do you think that providing forecasts regarding the evolution of indoor air quality improves the user experience?	31	2

## Data Availability

Data are contained within the article.

## Abbreviations

Concept	Abbreviation
Air Quality Index	AQI
Heating, Ventilation, and Air Conditioning	HVAC
Carbon Dioxide	CO_2_
Carbon Monoxide	CO
Building Management System	BMS
Constrained Application Protocol	CoAP
Hypertext Transfer Protocol	HTTP
Indoor Air Quality	IAQ
Internet of Things	IoT
Lightweight Machine to Machine	LwM2M
Message Queuing Telemetry Transport	MQTT
Not Only SQL	NoSQL
Nitrogen Dioxide	NO_2_
Nitrogen Oxides	NOx
Oxygenated Volatile Organic Compounds	OVOC
Ozone	O_3_
Particulate Matter	PM
Sick Building Syndrome	SBS
Structured Query Language	SQL
Sulfur Dioxide	SO_2_
Total Volatile Organic Compounds	TVOC
Volatile Organic Compounds	VOC
